# miRNA-543 attenuates placental angiogenesis in female fetal growth restriction by targeting ANGPT2 and inhibiting the PI3K/AKT/mTOR pathway

**DOI:** 10.3389/fcell.2026.1912518

**Published:** 2026-10-16

**Authors:** Wenhui Song, Xuemei Yang, Chunyan Yang, Yuanyuan Zhao, Xinxian Zhang, Qing Guo

**Affiliations:** 1 Clinical Medicine Research Center, The Fourth Hospital of Shijiazhuang, Shijiazhuang, Hebei, China; 2 Department of Obstetrics, The Fourth Hospital of Shijiazhuang, Shijiazhuang, Hebei, China; 3 Department of Obstetrics, Xingtai People’s Hospital, Xingtai, Hebei, China; 4 Institute of Reproductive Medicine of Shijiazhuang, The Fourth Hospital of Shijiazhuang, Shijiazhuang, Hebei, China; 5 Hebei Key Laboratory of Maternal and Fetal Medicine,The Fourth Hospital of Shijiazhuang, Shijiazhuang, Hebei, China

**Keywords:** angiopoietin 2, apoptosis, endothelial cell, fetal growth retardation, microRNA, placenta

## Abstract

**Background:**

Insufficient placental angiogenesis is a predominant pathological cause of fetal growth restriction (FGR). Placental capillary development is regulated in a sex-dependent manner, with female placentas exhibiting greater susceptibility to angiogenic dysfunction than male placentas. However, the sex-specific interplay between microRNAs (miRNAs) and human placental angiogenesis in FGR pathogenesis remains poorly defined. Emerging transcriptomic sequencing evidence has implicated miR-543 as a pivotal regulator of impaired angiogenesis in female FGR placentas. This study aimed to elucidate the sex-dependent mechanistic role of miR-543 in modulating placental angiogenesis in a cell-specific manner.

**Method:**

FGR pregnancies were recruited, and placental tissues were collected after delivery. The dual-luciferase reporter assay was used to detect the direct binding site of miR-543 to ANGPT2. Next, immunohistochemistry (IHC) and immunofluorescence (IF) staining were used to detect the expression and localization of ANGPT2. To verify the cell-specific gene expression of miR-543 and ANGPT2, laser capture microdissection (LCM) was employed to isolate endothelial cells from human placentas. The gene expression levels of miR-543 and ANGPT2 were quantified by qRT-PCR. CCK8, wound healing, Transwell, and tube formation assays and flow cytometry were performed to assess the functional effects of miR-543 and ANGPT2 on the biological function of human umbilical vein endothelial cells (HUVECs). The PI3K/AKT/mTOR signaling pathway was predicted and verified by Western blotting.

**Results:**

Significantly downregulated miR-543 expression was observed in the placentas of female FGR fetuses relative to matched normal controls. According to target gene prediction and the dual-luciferase reporter assay, ANGTP2 was identified as a target candidate of miR-543. Moreover, immunofluorescence staining and LCM analysis verified the cell-specific distribution of ANGPT2 in female FGR placentas, partly indicating its key involvement in placental capillary angiogenesis. Furthermore, dysregulated expression of miR-543 and ANGPT2 was closely correlated with aberrant proliferation, migration, and apoptosis of HUVECs. KEGG analysis revealed that the candidate targets of miR-543 were mainly enriched in the PI3K/AKT/mTOR pathway. Western blot analysis demonstrated that the PI3K/AKT/mTOR signaling pathway mediated the ANGPT2-induced attenuation of placental angiogenesis in female FGR.

**Conclusion:**

miR-543 negatively regulated ANGPT2 expression, leading to impaired endothelial cell function via inactivation of the PI3K/AKT/mTOR signaling pathway. This, in turn, contributed to sex-specific inhibition of capillary angiogenesis in the placenta of female fetuses. These findings reveal a novel miRNA-mediated, sex-dependent regulatory mechanism underlying the pathogenesis of impaired placental angiogenesis.

## Introduction

Fetal growth restriction (FGR), affecting 5%–10% of pregnancies, is a common pregnancy complication, characterized by a fetus that fails to achieve its genetic growth potential. It is associated with adverse perinatal outcomes, including increased morbidity and mortality for both mothers and infants, as well as adverse long-term health consequences for affected offspring (Tun et al., 2019). The pathophysiology of FGR is predominantly attributed to placental insufficiency resulting from inadequate vascular development (Gumina and Su, 2023). The non-branching phase of placental angiogenesis is widely recognized as a critical period for establishing the villous microvasculature—the functional unit of fetoplacental exchange (Wang et al., 2022). Proper microvascular architecture mainly relies on the precise orchestration of endothelial cell (EC) functions, including proliferation, migration, and survival. Dysregulated apoptosis of endothelial cells directly leads to the collapse or developmental arrest of the placental microvascular network, thereby contributing to placental insufficiency.

Growing concerns focus on the sex-dependent effect in FGR pregnancy due to the discrepant growth traits between male and female fetuses in the uterus (Christians and Chow, 2022). Some research studies have suggested that female babies are more likely to have reduced sensitivity to prenatal stress (Xu et al., 2007). However, male fetuses may have greater susceptibility to early-life effects but be less susceptible to insults in the uterus (Li et al., 2013; Sutherland and Brunwasser, 2018). Few studies have demonstrated the role of epigenetic regulation in placental angiogenesis in a sex-dimorphic manner.

Emerging evidence highlights non-coding RNAs, particularly miRNAs, as key epigenetic regulators of placental angiogenesis (Liu L. et al., 2024). Our previous study found significantly lower capillary density in female FGR placenta than in the control group and primarily identified miR-543 as a potential regulator of placental angiogenesis (Song et al., 2024). Although miR-543 has been implicated in endothelial cell (EC) survival and tube formation in cancer models (Liu L. et al., 2024), the precise downstream targets and functional pathways through which it orchestrates this sex-dimorphic angiogenic impairment in placental development remain entirely unknown.

To address this gap, we performed *in silico* target prediction and bioinformatics pathway analysis to screen for downstream effectors of miR-543 and examined how these targets and their signaling cascades regulate EC-mediated angiogenesis. This study aimed to uncover a novel regulatory axis of miR-543 in female FGR, offering mechanistic insights into sex-specific placental angiogenesis.

## Methodology

### Human placenta tissue collection

Placental tissue samples were collected from pregnant women who underwent vaginal delivery at the Fourth Hospital of Shijiazhuang between January 2022 and December 2024. All recruited pregnancies provided informed consent. The participants were categorized into an FGR group, defined by a birth weight below the 10th percentile for gestational age (American College of Obstetricians and Gynecologists’ Committee on Practice Bulletins—Obstetrics and The Society for Maternal-Fetal Medicine, 2019), and a control group comprising pregnancies with normal birth weight, with each group consisting of 10 male and female participants. The exclusion criteria were as follows: 1) pregnancy-related complications, such as preeclampsia or gestational diabetes; 2) gestational age less than 37 weeks; and 3) multiple pregnancies. The characteristics of the cases are listed in Supplementary Table S1. This study was approved by the hospital ethics committee (Approval ID: 20210030) and registered with the Chinese Clinical Trial Registry (Registration Number: ChiCTR2100043159) on 7th February 2021. Placenta tissue samples were retrieved within 30 min after vaginal delivery, frozen in liquid nitrogen, and stored at −80 °C for RNA analysis. For immunohistochemistry and immunofluorescence analyses, placental fragments were fixed overnight at 4 °C in 4% paraformaldehyde and embedded in paraffin or frozen in liquid nitrogen. To avoid discrepancies, small fragments of placental tissues were collected from five different regions of the placenta. Additionally, each fetoplacental tissue sample was collected from a site 4–6 cm away from the umbilical cord insertion.

### Bioinformatic prediction and dual-luciferase reporter assay

Candidate targets of miR-543 were predicted using TargetScan 7.2 and miRanda 5.0, and only genes identified by both algorithms were considered for further analysis. Functional enrichment analysis, including GO terms and KEGG pathways, was performed on this overlapping gene set.

To validate the predicted miR-543 binding site in the 3′UTR of ANGPT2, we generated two reporter plasmids: hsa_ANGPT2_miR543-wt (containing the wild-type binding sequence) and hsa_ANGPT2_miR543-mut (carrying a mutated seed sequence). HEK-293T cells were seeded in 24-well plates and co-transfected with the reporter plasmid and either miR-543 mimics or negative control mimics (mimics-NC), all obtained from GenePharma Co., Ltd. At 24 h post-transfection, cells were lysed, and firefly luciferase activity was measured and normalized to Renilla luciferase activity according to the manufacturer’s instructions (Dual-Luciferase Reporter Gene Assay Kit, GenePharma Co., Ltd.).

### Immunohistochemistry and immunofluorescence staining of ANGPT2 in placental villi

Paraffin-embedded human placental tissues were sectioned at 4 μm and automatically stained with a rabbit monoclonal anti-human ANGPT2 primary antibody using the ultraView Universal DAB Detection Kit (Ventana Medical Systems, Tucson, AZ, United States) on a VENTANA BenchMark GX® (Roche, Tucson, AZ, United States) staining system according to the manufacturer’s protocol. Digital slides were scanned at 40× magnification using an Aperio CS2 scanner (Aperio Technologies Inc., San Diego, CA, United States). For each section, five equal-sized fields enriched in microvessels were selected by an experienced pathologist. Staining density was quantified by color deconvolution using Aperio ImageScope software (Aperio Technologies Inc.).

OCT-embedded frozen human placenta tissues were sectioned at 8 μm. After removing the OCT compound, sections underwent antigen retrieval and blocking with 3% H_2_O_2_ and 5% BSA. Sections were incubated overnight at 4 °C with primary antibodies against CD31 (mouse, 28083-1-AP, Proteintech, China) and ANGPT2 (rabbit, 1:1,000, ab199133, Abcam). Secondary antibodies, goat anti-mouse IgG (DyLight 488, A23210, Abbkine) and goat anti-rabbit IgG (DyLight 649, A23620, Abbkine), were applied for 1 h at room temperature. Nuclei were counterstained with DAPI. Images were acquired using an Olympus FV3000 confocal microscope.

### Co-localization of miR-543 and CD31 via FISH and IF

Dual staining of miR-543 and CD31 was carried out on frozen sections of human placenta using fluorescence *in situ* hybridization (FISH) combined with immunofluorescence (IF). In brief, FISH was conducted using a Cy3-labeled miR-543 probe (5′-AAGAAGTGCACCG CGAATGTTT-3′) and a FISH kit (GenePharma, Shanghai, China) according to the manufacturer’s instructions. Following post-hybridization SSC washes, sections were blocked with 5% BSA (Beyotime, Beijing, China) for 1 h at room temperature (RT) and incubated overnight at 4 °C with an anti-CD31 primary antibody (1:200, 28083-1-AP, Proteintech). Sections were then incubated with corresponding secondary antibodies for 1 h at RT, counterstained with DAPI for 10 min, and imaged using a confocal laser scanning microscope (TCS SP8; Leica Microsystems, Wetzlar, Germany).

### Laser capture microdissection

Frozen human placenta sections (12 μm) mounted on PEN membrane slides were stained with hematoxylin and eosin (H&E). After dehydration, the slides were placed on the microscope stage, and capillary-rich regions were identified for microdissection. Endothelial cells within these regions were captured using the ArcturusXT™ LCM System (Life Technologies Corporation, Carlsbad, CA, United States) with CapSure® HS caps, strictly following the manufacturer’s instructions (Krieger et al., 2012). The caps containing the microdissected cells were then transferred to microcentrifuge tubes for subsequent RNA extraction. The LCM procedure is illustrated in Supplementary Figure S1.

### Reverse transcription quantitative polymerase chain reaction

Total RNA was extracted from cultured cells and tissue samples using the Eastep® Super Total RNA Extraction Kit (Shanghai Promega, China), while microRNA was isolated using the miRNeasy Mini Kit (QIAGEN, 217004). For LCM-derived samples, RNA was extracted using the PicoPure RNA Isolation Kit (Catalog # KIT0202/KIT0204, Applied Biosystems, United States). Reverse transcription was performed using the SuperScript® III First-Strand Synthesis System (Catalog # 18080-051, Invitrogen, United States). Quantitative PCR was conducted on a real-time PCR system using SYBR Green PCR Kits (RiboBio Co., Ltd., Guangzhou, China) for both mRNA and miRNA detection. The relative expression levels were normalized to GAPDH (for mRNA) or U6 (for miRNA) and calculated using the 2^−ΔΔCT^ method. The primer sequences for GAPDH were 5-GTC​TCC​TCT​GAC​TTC​AAC​A GCG-3 (forward) and 5-ACC​ACC​CTG​TTG​CTG​TAG​CCA​A-3 (reverse); for ANGPT2, the primers were 5-ATT​CAG​CGA​CGT​GAG​GAT​GGC​A-3 (forward) and 5-GCACATAGCGTT GCTGATTAGTC-3 (reverse). miRNA-specific primers were purchased from RiboBio (Guangzhou, China). All primer pairs exhibited amplification efficiencies between 90% and 115%, and melting curve analysis confirmed the specificity of each amplification.

### Cell culture and cell transfection

The human umbilical vein endothelial cell (HUVEC) line was obtained from ScienCell Research Laboratories (Carlsbad, California, United States). HUVECs were cultured in a constant-temperature incubator at 37 °C with 5% CO_2_ in endothelial cell complete medium (ECM), which contained 10% fetal bovine serum (FBS) and 1% penicillin–streptomycin solution. Cell growth was monitored, and the complete medium was routinely replaced.

### Transfection of siRNA and miRNA

HUVECs were seeded in 6-well plates at 1 × 10^5^ cells/well and transfected with ANGPT2 siRNA (50 nM), the ANGPT2 overexpression plasmid, miR-543 mimics, or miR-543 inhibitors using Lipofectamine 2000 (Invitrogen, United States). Corresponding empty vectors or negative control oligonucleotides (GenePharma, Suzhou, China) served as controls. Transfection efficiency was evaluated by RT-qPCR. Cells were harvested at 72 h after plasmid transfection or 48 h after oligonucleotide transfection. For rescue experiments, the PI3K inhibitor LY294002 was added. Primer and oligonucleotide sequences are detailed in Supplementary Table S2.

### Cell counting kit-8 assay

Cell proliferation was evaluated using the CCK-8 assay (Beyotime, Shanghai, China). HUVECs were seeded in 96-well plates at 5 × 10^3^ cells/well in 100 μL medium, with five replicate wells per group. At 0, 24, 48, and 72 h post-seeding, cells were washed twice with PBS (Gibco, Thermo Fisher, United States), and 10 μL of CCK-8 solution was added to each well in fresh medium. Absorbance was measured at 450 nm using a microplate reader (Bio-Rad, United States).

### Wound healing migration assay

Cell migration was assessed using a scratch assay. HUVECs were seeded in 6-well plates at 5 × 10^5^ cells/well. A straight scratch was created across each well using a 200 μL pipette tip. Detached cells were removed by washing twice with PBS, and the cells were then cultured in serum-reduced medium. Wound images were captured at 0, 1, 2, and 6 h using an Olympus IX71 inverted microscope. The wound closure rate was calculated as the percentage of the healed area relative to the initial wound area.

### Transwell migration and invasion assay

Cell migration and invasion were evaluated using Transwell systems (Corning, NY, United States). Following transfection (48 h for oligonucleotides or 72 h for plasmids), HUVECs were resuspended in serum-free ECM at a concentration of 2.5 × 10^5^ cells/mL. For migration assays, 200 μL of cell suspension was added to the upper chamber, and 800 μL of ECM containing 20% FBS was placed in the lower chamber. After 24 h of incubation at 37 °C, cells on the lower membrane surface were fixed with 4% paraformaldehyde (Solarbio, China) and stained with 0.1% crystal violet (Solarbio, China). Stained cells were counted in five randomly selected fields per membrane using an Olympus microscope (Japan). For invasion assays, the procedure was identical, except that the upper chamber membrane was pre-coated with Matrigel (diluted in serum-free ECM and solidified for 4 h at 37 °C) before cell seeding.

### Tube formation assay

Tube formation ability was assessed using a Matrigel-based angiogenesis assay. Matrigel was thawed overnight at 4 °C, and pre-chilled 96-well plates and tips were prepared. Matrigel was diluted 1:8 with ECM, and 60 μL of the diluted Matrigel was added to each well, avoiding bubble formation. The plate was incubated at 37 °C for 30 min to allow gelation. HUVECs were seeded at an optimized density of 2 × 10^4^ cells per well. After 24 h of incubation, cells were stained with 100 μL of calcein AM per well for 30 min at 37 °C. Tubules were visualized using a fluorescence microscope with a FITC filter.

### Flow cytometry and tunnel assay

Cell apoptosis was assessed via flow cytometry using the Apoptosis Detection Kit (BD Biosciences, United States) following the manufacturer’s instructions. In brief, treated cells were harvested, washed with cold PBS, and resuspended in 1 × binding buffer. Cells were stained with Annexin V-PE and 7-AAD in the dark for 15 min at room temperature. Flow cytometric data were acquired on a flow cytometer (Beckman Coulter, United States) and analyzed using FlowJo software (version 10.8). A detailed description of the sequential three-step gating strategy is presented in Supplementary Figure S3. The cell apoptotic rate was calculated as the ratio of early and late apoptotic cells to the total number of cells.

At 70%–80% confluence, cell apoptosis induced rescue experiment was evaluated using a One-Step TUNEL Assay Kit (Abbkine) according to the manufacturer’s protocol.

### Western blotting

Protein expression was assessed by Western blotting. Total protein was extracted from HUVECs and fetoplacental tissues using RIPA lysis buffer (Solarbio) containing 1% PMSF. Equal amounts of protein were separated by 10% SDS-PAGE (Solarbio) and transferred onto PVDF membranes (0.22 μm, Millipore, United States). The membranes were blocked with 5% skim milk for 1 h at room temperature and then incubated with primary antibodies overnight at 4 °C. The following primary antibodies were used: ANGPT2 (ab199133, 1:1,000, Abcam), caspase 3 (1:100, Santa Cruz), GAPDH (1:5,000, Jingjie PTM BioLab), ERK (1:1,000, ZEN-BIOSCIENCE), p-ERK (1:2000, Cell Signaling Technology), AKT (1:1,000, ZEN-BIOSCIENCE), p-AKT (1:2000, Cell Signaling Technology), mTOR (1:1,000, ZEN-BIOSCIENCE), and p-mTOR (1:1,000, ZEN-BIOSCIENCE). After incubation with HRP-conjugated secondary antibodies (goat anti-rabbit IgG, 1:5,000; goat anti-mouse IgG, 1:5,000, Jingjie PTM BioLab) for 2 h at room temperature, protein bands were visualized using an enhanced chemiluminescence reagent (Share Bio, Shanghai, China) and quantified using ImageJ software. To investigate the underlying mechanism, rescue experiments with the PI3K inhibitor LY294002 were also performed to confirm pathway specificity.

### Statistical analysis

Statistical analyses were performed using GraphPad Prism 7.0 and SPSS 26.0. Data are presented as the mean ± SD. For comparisons between two groups, Student’s t-test was applied to normally distributed data with equal variances; otherwise, the Mann–Whitney U test was used. One-way ANOVA was used for the rescue experiment, and two-way ANOVA, followed by Sidak’s multiple comparisons test, was applied for CCK-8 data. Categorical variables were analyzed using the chi-square test or Fisher’s exact test. All experiments were independently repeated three times. A *p-*value < 0.05 was considered statistically significant.

## Results

### Validation of sex-dependent expression of miR-543 in FGR placenta

Baseline demographic characteristics were well-matched between the FGR and control groups (Supplementary Table S1). Consistent with our previous sequencing data identifying miR-543 as the most significantly dysregulated miRNA in female FGR placentas (Song et al., 2024), qRT-PCR validation confirmed a marked downregulation of miR-543 in female FGR placentas compared with controls (*p* = 0.008) (Figure 1A). No significant difference was observed in male FGR placentas compared with male controls (*p* = 0.126). These findings confirm sex-specific dysregulation of placental miR-543 in FGR. Dual FISH/IF staining of frozen placental sections further revealed co-localization of miR-543 with CD31-positive endothelial cells in female placentas (Figure 1B).

**FIGURE 1:**
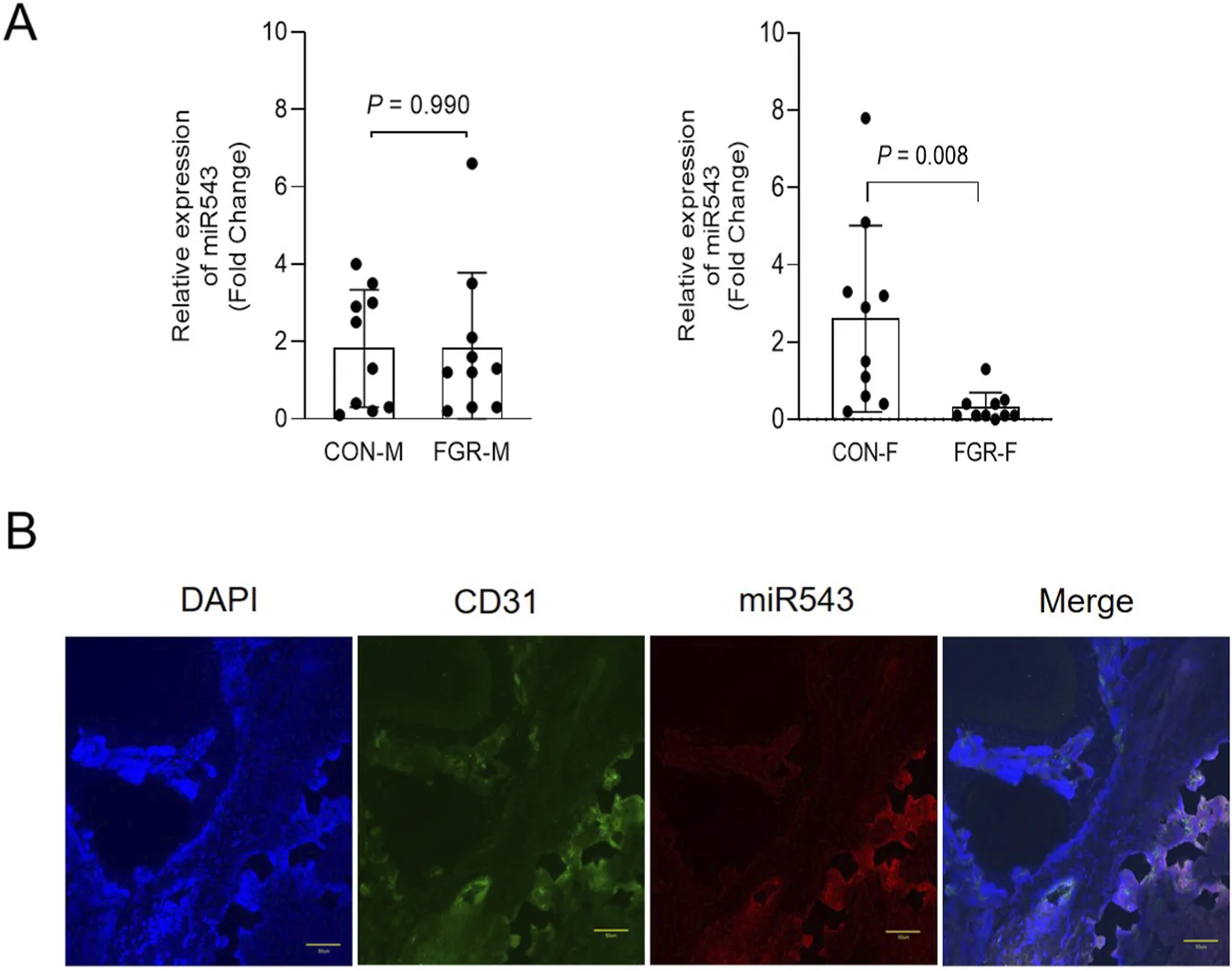
Gene expression and location of miR-543 in the female placenta. **(A)** Relative gene expression of miR-543 in male and female placentas. **(B)** Co-localization of CD31 and miR-543 in the female placenta. Data are expressed as the mean ± SD, n = 10. Statistical significance was defined as *p* < 0.05.

### Effect of miR-543 on the disruption of HUVEC proliferation, cell immigration, invasion, and tube formation

To investigate the functional role of miR-543 in HUVECs, cells were transfected with miR-543 mimics or inhibitors. qRT-PCR confirmed significant upregulation and downregulation of miR-543 expression in the respective groups compared with negative controls (Figure 2A).

**FIGURE 2:**
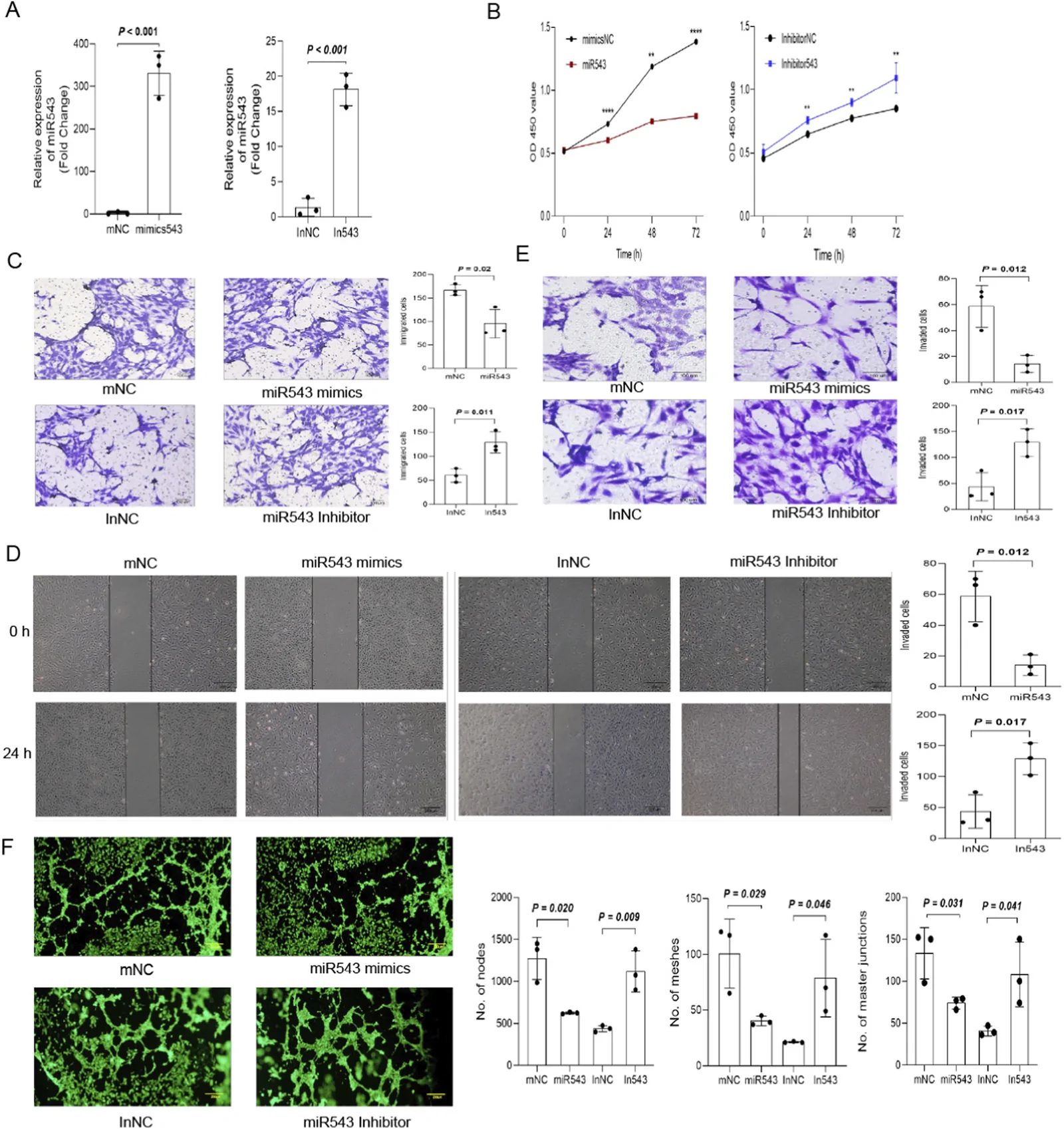
Effects of miR-543 on HUVEC proliferation, migration, invasion, and tube formation. **(A)** qRT-PCR validation of miR-543 expression after transfection with miR-543 mimics or inhibitors. **(B)** CCK-8 assay of HUVEC proliferation at 0, 24, 48, and 72 h post-transfection. **(C)** Transwell migration assay (scale bar: 100 μm). **(D)** Wound healing assay (scale bar: 200 μm). **(E)** Transwell invasion assay (scale bar: 50 μm). **(F)** Tube formation assay stained with calcein AM (scale bar: 100 μm). Data are presented as the mean ± SD from three independent experiments (n = 3 per group). Statistical significance was defined as *p* < 0.05.

CCK-8 assays showed that miR-543 overexpression reduced OD450 values over time, whereas miR-543 inhibition increased proliferation relative to controls (Figure 2B). Two-way ANOVA revealed significant effects of treatment, time, and their interaction, indicating a time-dependent inhibitory effect of miR-543 on proliferation.

Transwell migration and invasion assays demonstrated that miR-543 knockdown significantly increased cell migration and invasion, while miR-543 overexpression exerted opposite effects (Figures 2C,E). These findings were corroborated by wound healing assays (Figure 2D).

Tube formation assays further revealed that miR-543 mimics significantly reduced node number, master junctions, meshes, and total tube length, whereas miR-543 inhibitors enhanced these angiogenic parameters (Figure 2F). Collectively, these results indicate that miR-543 negatively regulates HUVEC proliferation, migration, invasion, and angiogenesis.

### ANGPT2 was a target of miR-543

To identify potential downstream targets of miR-543, bioinformatics prediction was performed using TargetScan and miRanda. The intersection of the two databases yielded ANGPT2 and AGGF1 as candidate targets, both of which are pro-angiogenic factors. ANGPT2 was prioritized for further study based on its higher predictive confidence scores (miRanda energy score: −11.6; TargetScan score: 95) (Table 1).

**TABLE 1:** Target gene prediction of hsa-miR-543.

miRNA ID	Symbol	TargetScan_score	miranda_energy
hsa-miR-543	ANGPT2	95	−11.6
hsa-miR-543	AGGF1	94	−11.32

Direct binding between miR-543 and the 3′-UTR of ANGPT2 mRNA was validated by a dual-luciferase reporter assay (Supplementary Figure S2). Co-transfection of miR-543 mimics with the wild-type ANGPT2 reporter plasmid significantly reduced luciferase activity compared with controls (*p* < 0.001), whereas no significant change was observed with the mutant reporter plasmid (*p* > 0.05). These results confirm that miR-543 directly and specifically binds to the 3′-UTR of ANGPT2 mRNA.

### Localization and expression profiling of ANGPT2 in female FGR placenta

Immunofluorescence staining revealed predominant co-localization of ANGPT2 with the endothelial marker CD31 in human placental tissues (Figure 3A). Quantitative immunohistochemical analysis further demonstrated a significant increase in ANGPT2 expression in placental tissues from female FGR cases compared with controls (Figure 3B).

**FIGURE 3:**
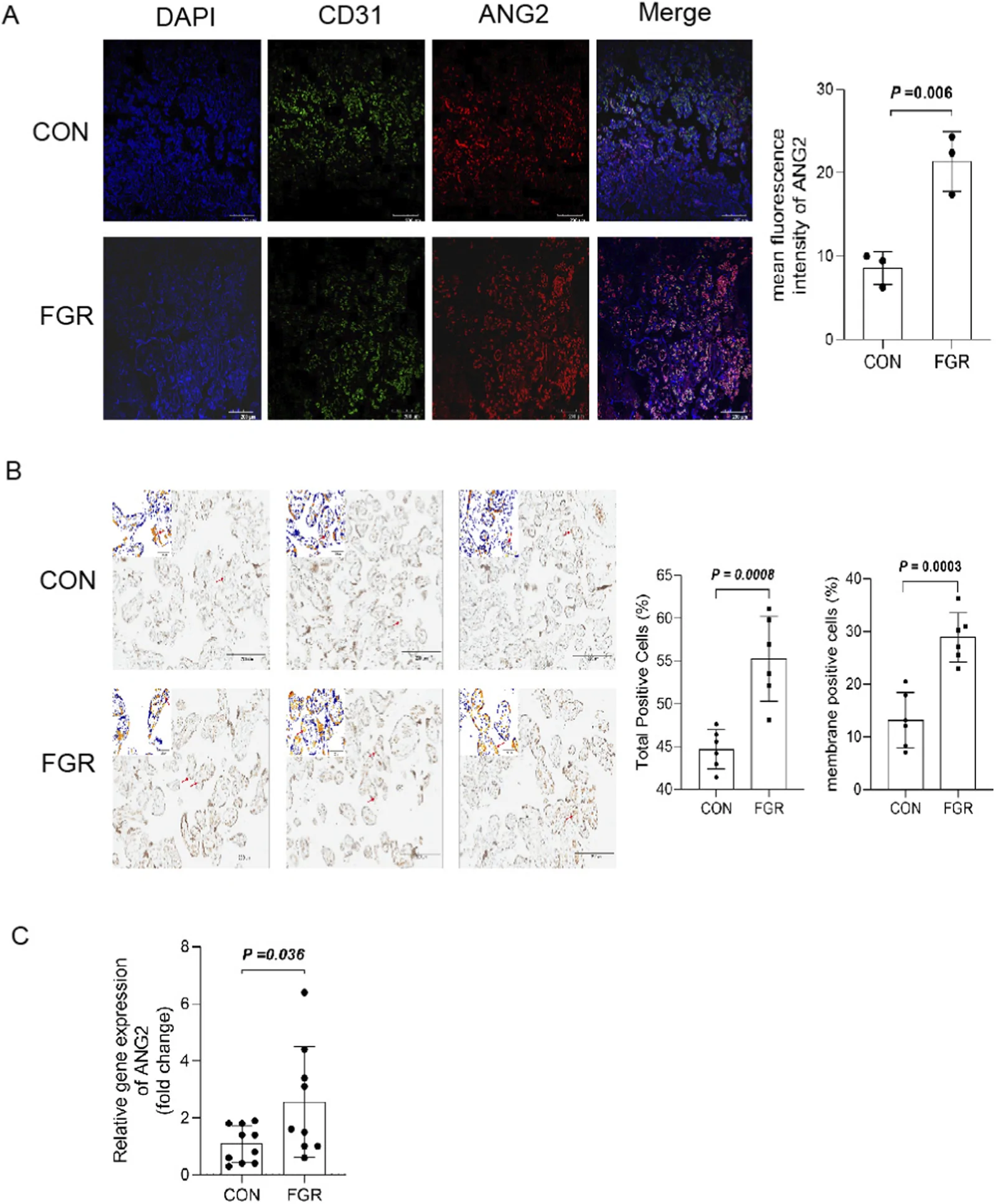
Localization and expression of ANGPT2 in female placental tissues. **(A)** Immunofluorescence staining of ANGPT2 (red), CD31 (green), and DAPI (blue) in normal and FGR placentas. Scale bar: 200 μm. Relative fluorescence intensity of ANGPT2 was compared between groups (n = 10). **(B)** Immunohistochemical staining of ANGPT2 in normal and FGR placental tissues. Scale bar: 200 μm. Insets (left upper corner) show color deconvolution-transformed images. Scale bar: 50 μm. Red arrows indicate stained cells around capillaries. Total positive rate and membrane positive rate are presented (n = 6). **(C)** Relative ANGPT2 mRNA expression in LCM-isolated endothelial cells from placental frozen sections (n = 10). Data are presented as the mean ± SD from three independent experiments. Statistical significance was defined as *p* < 0.05.

To validate endothelial-specific expression at the cellular level, LCM was used to isolate endothelial cells from frozen placental sections. qRT-PCR analysis of isolated endothelial cells confirmed a marked upregulation of ANGPT2 expression in the FGR group (Figure 3C), consistent with the tissue-level findings.

### ANGPT2 promotes HUVEC proliferation, migration, and tube formation, and inhibits apoptosis

To assess the functional role of ANGPT2 in endothelial cells, HUVECs were transfected with ANGPT2 overexpression plasmids or siRNAs. RT-qPCR confirmed successful modulation of ANGPT2 expression (Figure 4A).

**FIGURE 4:**
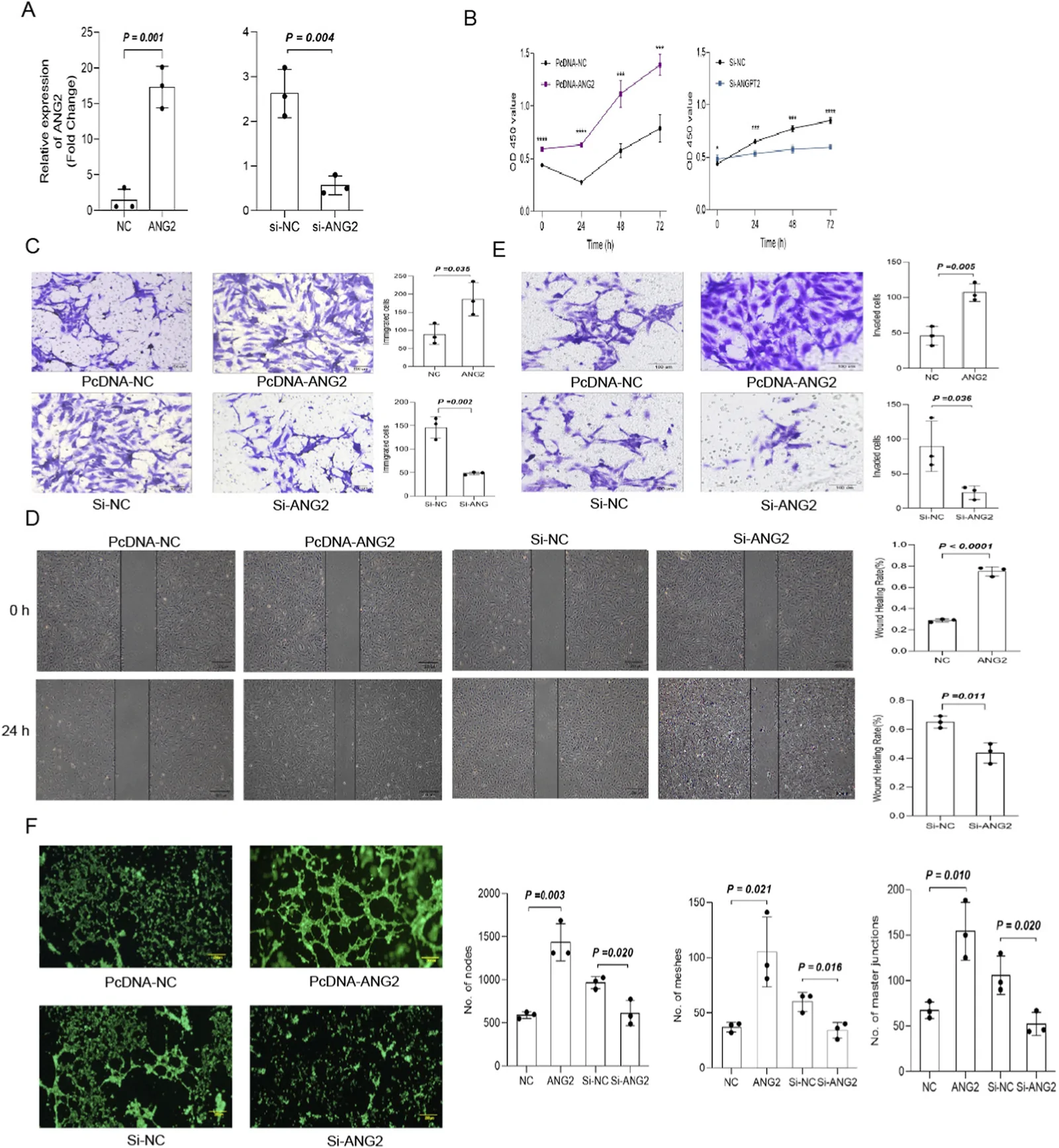
Effects of ANGPT2 on HUVEC proliferation, migration, invasion, and tube formation. **(A)** qRT-PCR validation of ANGPT2 expression after transfection with pcDNA-ANGPT2 or si-ANGPT2. **(B)** CCK-8 assay of HUVEC proliferation at 0, 24, 48, and 72 h post-transfection. **(C)** Transwell migration assay (scale bar: 100 μm). **(D)** Wound healing assay. **(E)** Transwell invasion assay. **(F)** Tube formation assay stained with calcein AM (scale bar: 100 μm). Data are presented as the mean ± SD from three independent experiments (n = 3 per group). Statistical significance was defined as *p* < 0.05.

CCK-8 assays showed that ANGPT2 overexpression promoted HUVEC proliferation, whereas ANGPT2 knockdown attenuated proliferation (Figure 4B). Transwell migration and wound healing assays further demonstrated that ANGPT2 overexpression enhanced cell migration, while ANGPT2 knockdown impaired migration (Figures 4C,D). Similarly, invasion capacity was increased upon ANGPT2 overexpression and reduced upon ANGPT2 knockdown (Figure 4E).

Tube formation assays revealed that ANGPT2 depletion significantly impaired angiogenic capacity (Figure 4F). Collectively, these results indicate that ANGPT2 positively regulates HUVEC proliferation, migration, invasion, and angiogenesis.

### miR-543 facilitates HUVEC apoptosis via targeted repression of ANGPT2

Flow cytometry with PE-Annexin V/7-AAD staining showed that miR-543 overexpression significantly increased HUVEC apoptosis, whereas its knockdown reduced apoptosis (Figure 5). Conversely, ANGPT2 overexpression decreased apoptosis, while ANGPT2 knockdown promoted it (Figure 5). Collectively, these findings confirm that miR-543 promotes HUVEC apoptosis by negatively regulating ANGPT2.

**FIGURE 5:**
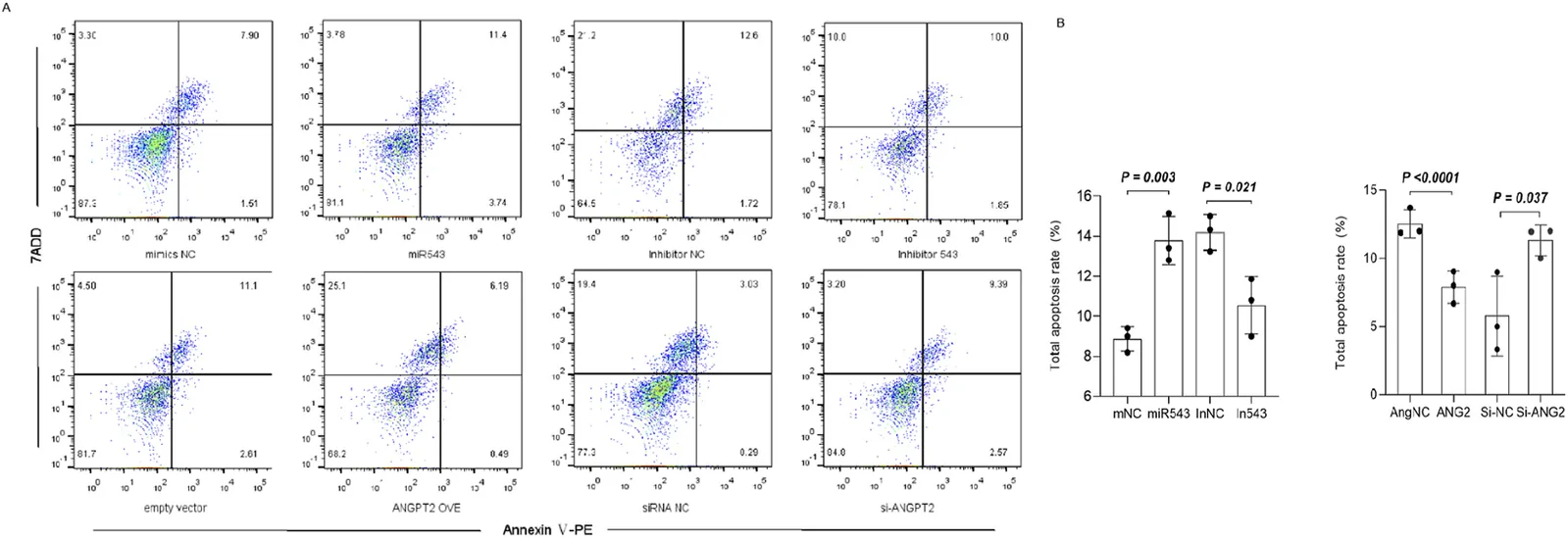
Apoptosis analysis of HUVECs by flow cytometry. **(A)** Representative flow cytometry dot plots showing viable (lower left), early apoptotic (lower right), and late apoptotic/necrotic (upper right) cells stained with Annexin V-FITC and PI. **(B)** Quantification of early and late apoptotic cells from three independent experiments. Data are presented as the mean ± SD (n = 3 per group). Statistical significance was defined as *p* < 0.05.

### miR-543 targeting ANGPT2 induces apoptosis of HUVECs via the PI3K/AKT/mTOR signaling pathway

Western blotting was performed to examine the regulatory effects of the miR-543/ANGPT2 axis on apoptotic and signaling proteins (Figure 6A). miR-543 inhibition upregulated ANGPT2 protein expression, whereas miR-543 overexpression downregulated ANGPT2, confirming a negative regulatory relationship. Caspase-3 expression was increased upon miR-543 overexpression or ANGPT2 silencing, consistent with the apoptosis phenotypes.

**FIGURE 6:**
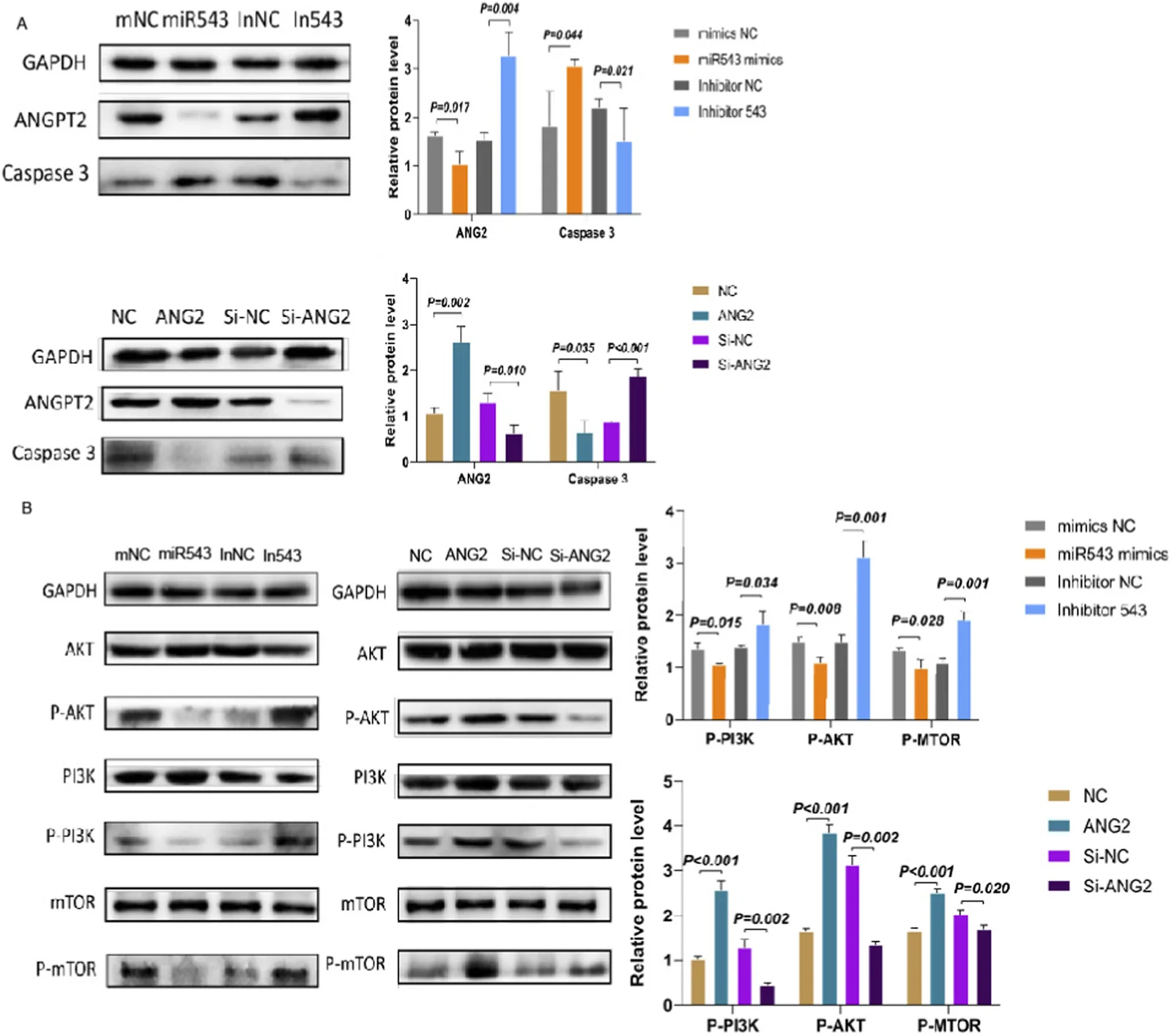
miR-543/ANGPT2 axis regulates HUVEC apoptosis via the PI3K/AKT/mTOR signaling pathway. **(A)** Protein expression of ANGPT2 and caspase-3 in transfected groups. **(B)** Phosphorylation levels of PI3K, AKT, and mTOR in transfected groups. Data are presented as the mean ± SD from three independent experiments. Statistical significance was defined as *p* < 0.05.

To explore the underlying mechanism, GO and KEGG enrichment analyses were performed based on miR-543 target genes. KEGG analysis predicted enrichment of the PI3K/AKT pathway in female FGR placentas (Supplementary Figure S4). Western blotting confirmed that phosphorylation levels of PI3K and AKT were suppressed by miR-543 overexpression or ANGPT2 knockdown, whereas they were increased by miR-543 inhibition or ANGPT2 overexpression (Figures 6B, 7B). mTOR phosphorylation was similarly enhanced upon miR-543 downregulation or ANGPT2 overexpression and reduced upon miR-543 overexpression with ANGPT2. Treatment with LY294002 enhanced apoptosis, confirming the involvement of the PI3K/AKT pathway (Figure 7A). Consistently, the TUNEL assay showed that ANG2 reversed miR-543-induced apoptosis in a PI3K/AKT-dependent manner (Figure 7B).

**FIGURE 7:**
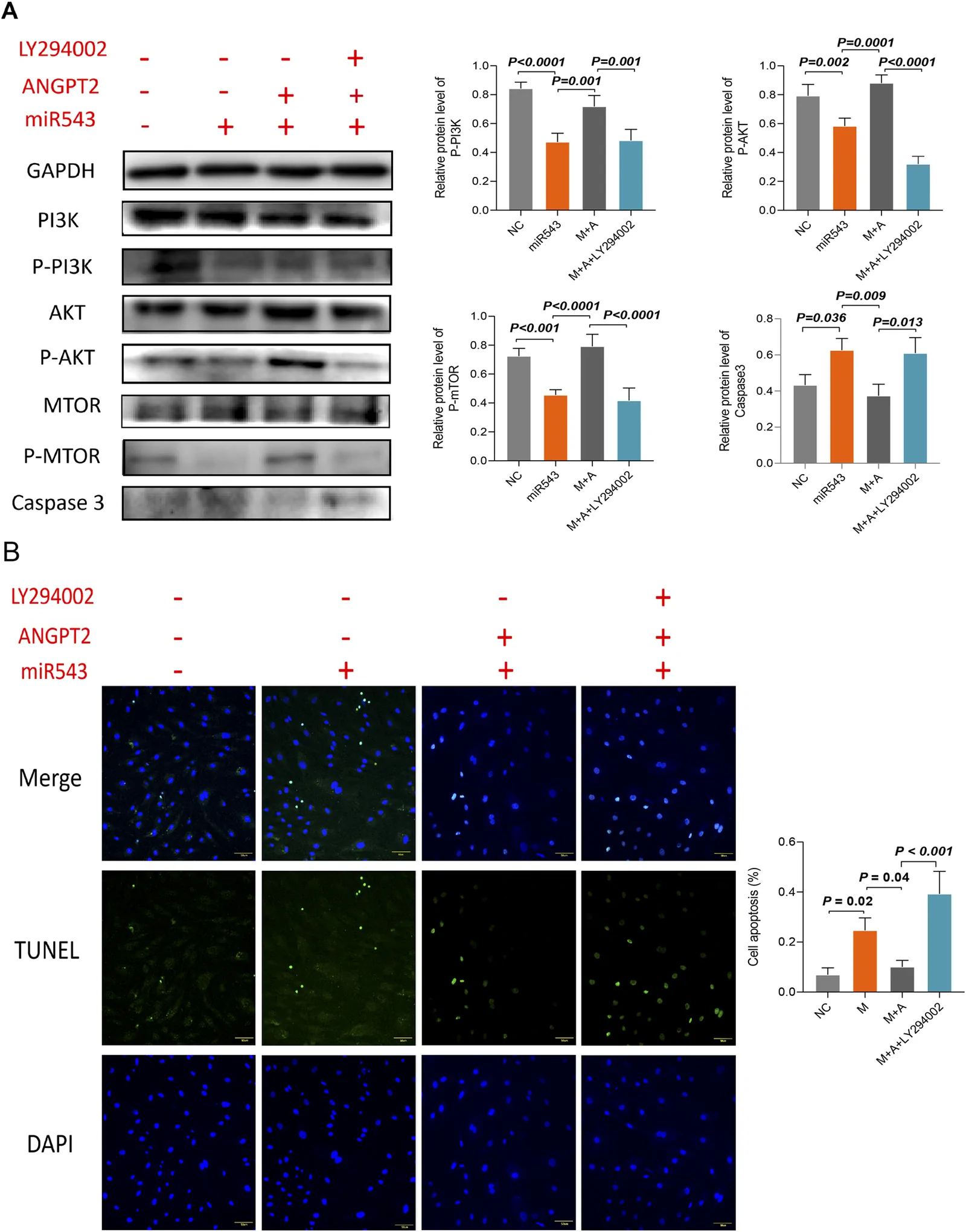
miR-543 targeting ANGPT2 regulates caspase-3 via the PI3K/AKT signaling pathway. **(A)** Cell apoptosis was detected using TUNEL assay. **(B)** The expressions of pathway-related proteins were detected using Western blotting. The experiments were repeated three times. Statistical significance was defined as *p* < 0.05.

Collectively, these results demonstrate that miR-543 directly targets and negatively regulates ANGPT2, thereby suppressing PI3K/AKT/mTOR signaling, inhibiting endothelial proliferation and angiogenesis, and promoting apoptosis. This miR-543/ANGPT2/PI3K/AKT/mTOR axis may represent a potential therapeutic target for female FGR.

## Discussion

Our findings reveal a fetal sex-dependent and cell-specific role of miR-543 in placental angiogenesis, supported by both *in vivo* and *in vitro* data. In female FGR placentas, miR-543 was downregulated, while its target ANGPT2 showed endothelial-specific expression and localization (as demonstrated with LCM and immunofluorescence). Mechanistically, miR-543 reduction enhanced HUVEC survival and suppressed apoptosis by activating the PI3K/AKT/mTOR pathway through targeting of ANGPT2.

### miR-543 as a potential compensatory regulator of placental angiogenesis in female FGR

Dysregulated placental angiogenesis is a prominent hallmark of fetal growth restriction (FGR), and accumulating evidence indicates that epigenetic mechanisms are deeply implicated in this pathological process (Shinohara et al., 2007). miR-543 has been reported to facilitate angiogenesis in multiple disease contexts, including non-small cell lung cancer (NSCLS) and myocardial infarction, where it modulates angiogenic activity of cardiac microvascular endothelial cells (Wang et al., 2020; Ke et al., 2019). Intriguingly, our results revealed an opposite role of miR-543 in human placental capillaries; decreased miR-543 exerted a pro-angiogenic effect in female placentas, indicating its compensatory role in female placental angiogenesis. This phenotype was consistent with observations in diabetic retinopathy, in which miR-543 suppresses angiogenic responses in human retinal endothelial cells (Ke et al., 2019).

Male fetuses were more susceptible to growth restriction than female offspring, highlighting inherent sex differences in placental epigenetic regulation and the compensatory capacity of the female placenta (Tekola-Ayele et al., 2019). In this study, we further validated the existence of a sex-specific miRNA profile in the FGR placenta, consistent with previous findings (Cvitic et al., 2020).

Furthermore, the miRNAs in placental endothelial cells could be modulated in a cell-specific manner (Zhou et al., 2023). Our findings revealed the localization of miR-543 in endothelial cell from the female FGR fetoplacental tissues using PCR of LCM-isolated cells and cellular immunofluorescence.

### miR-543 may suppress placental endothelial angiogenesis by targeting ANGPT2

miRNA–mRNA interactions play a vital role in the development of FGR by regulating cell function during placental development (Wu et al., 2023).

In this study, we verified that miR-543 directly binds to the 3′-UTR of human ANGPT2. This was further verified by the pro-angiogenic role of miR-543, which was mediated by targeting ANGPT2, as manifested by the positive changes in the biological activity of HUVECs. This direct interaction between miR-543 and ANGPT2 has also been validated in osteosarcoma models (Wang et al., 2017). In ovine placentomes, ANGPT2 expression was tightly correlated with capillary density (Shinohara et al., 2007), demonstrating its potentially role as an angiogenic marker linked to FGR (Zhou et al., 2025). It might play a complementary role in endothelial cell maturation due to its plastic state in response to stimuli associated with non-branching angiogenesis, acting as a critical regulator of biological behavior (Ochoa et al., 2001).

Given that ANGPT2 drove compensatory angiogenesis under hypoxic stress (Araujo Junior et al., 2017), we proposed that reduced miR-543 expression might serve as a biological indicator reflecting partial or complete placental adaptation to adverse intrauterine insults, an effect mediated by miR-543-dependent modulation of ANGPT2.

Immunolocalization analyses had previously shown that ANGPT2 was predominantly expressed in syncytiotrophoblasts and perivascular cells (Geva et al., 2002). Here, immunofluorescence staining of frozen placental sections confirmed the co-localization of ANGPT2 with the endothelial marker CD31. In line with the finding, transcript profiling of LCM-isolated cells indicated that ANGPT2 exerts its biological functions in a cell-specific manner, primarily targeting placental endothelial cells.

### miR-543 promotes endothelial cell apoptosis by targeting ANGPT2 via the PI3K/AKT/mTOR pathway

Existing literature has reported that miR-543 impairs trophoblast viability and induces trophoblast apoptosis via the HIF1A signaling cascade (Liu Q. et al., 2024). This observation further prompted an exploration of the influence of miR-543 on apoptosis in HUVECs. Functional assays revealed that miR-543 targeted ANGTP2 to trigger endothelial cell apoptosis through activation of the PI3K/AKT/mTOR signaling pathway. This regulatory axis was consistent with previous findings that ANGPT2 exerted an anti-apoptotic effect via PI3K signaling (Chen H. et al., 2023).

The PI3K/AKT/mTOR pathway was a core signaling cascade that modulated cell survival, growth, and proliferation (Middha et al., 2023). Mounting evidence has established that this pathway is critical for placental angiogenesis and that its aberrant activation or dysfunction contributed to the onset of pregnancy complications such as preeclampsia (Chen M. et al., 2023) and FGR (Xu et al., 2025). Consistently, robust evidence suggested that PI3K/AKT/mTOR activation conferred protection against cellular apoptosis, largely by upregulating the pro-survival gene Bcl-2 to suppress apoptotic signaling (Yan et al., 2020) and promote cell proliferation (Hassan et al., 2013). Moreover, pharmacological inhibition of the PI3K/AKT/mTOR pathway had been shown to abrogate the pro-viability effects of ANGPT2 in HUVECs (Yuan et al., 2018). Our experimental data recapitulated these findings, showing that inhibition of PI3K signaling markedly reduced caspase-3 expression in HUVECs, confirming that the PI3K/AKT/mTOR pathway mediated adaptive cytoprotective effects in endothelial cells.

## Limitations and strength

In summary, this study elucidated a novel regulatory mechanism whereby miR-543 targeted ANGPT2 to modulate impaired placental angiogenesis through the PI3K/AKT/mTOR pathway. Our findings strongly supported that miR-543 acts in a sex-dependent and cell-specific manner in placentas associated with female FGR. However, this study has several limitations. The relatively small sample size may restrict the statistical power. Moreover, HUVECs, although widely used as an endothelial model, may not fully recapitulate the biological features of primary placental endothelial cells, especially fetal placental microvascular endothelial cells. Finally, since the proposed mechanism was not validated *in vivo*, it may not able to elucidate the potential role of miR-543 or ANGPT2 as a therapeutic target for FGR treatment. Therefore, future studies using larger clinical cohorts, primary placental endothelial cells or placental organoids, and appropriate animal models are needed to further validate these findings.

## Data Availability

The original data presented in the study are included in the article or Supplementary Material. Any further inquiries can be directed to the corresponding author.
