# Supplementary material for: miRNA-543 attenuates placental angiogenesis in female fetal growth restriction by targeting ANGPT2 and inhibiting the PI3K/AKT/mTOR pathway
Source: Front Cell Dev Biol. 2026 Oct 16;14:1912518. doi: 10.3389/fcell.2026.1912518 (PMC13719480; doi:10.3389/fcell.2026.1912518)
Supplement: Supplementary file 1 [file Table1.doc]

Supplemental Table 1 Demographic of participants recruited for miRNA qPCR and LCM analysis

| **Category** | **AGA male**  **(n=10)** | **FGR male**  **(n=10)** | **AGA female**  **(n=10)** | **FGR female**  **(n=10)** |
| --- | --- | --- | --- | --- |
| Age(year) | 28.5(22-32) | 27.5(23-34) | 29.5(21-37) | 30.5(27-34) |
| Pre-pregnancy BMI(Kg/m^2^) | 19.8(18.1-26.0) | 20.2(17.0-27.6) | 22.7(16.5-26.3) | 18.8(16.3-26.2) |
| Delivery  Gestation  (week) | 39.43(38.57-40.43) | 39.64(38.00-40.43) | 39.07(38.14-40.43) | 39.72(38.14-40.57) |
| Birth weight  (g) | 3185  (3040-3620) | 2700^***a^  (2500-2800) | 3300  (3000-3800) | 2650***^b^  (2130-2850) |
| Pre-pregnancy weight(Kg) | 52(48-65) | 51.5(46-62) | 60(46-76) | 52.5(40-63) |
| Weight near term(Kg) | 69.5(65-85) | 69.5(60-76) | 74.5(60-89) | 63.75(55-75) |
| Weight gain(Kg) | 15.25(10-23) | 16.5(8-23) | 15(10-25) | 12.5(4-21) |
| Graduation  ⩾Bachelor’s | 6(60) | 4(40) | 3(30) | 7(70) |
| Graduation  Some college | 4(40) | 6(60) | 3(30) | 7(70) |

Data are median(range) for continuous dataor or number(rate) for categorical data. Data analysed by Student T test (continuous data) or Fisher’s exact test (categorical data). AGA appropriate for gestational age, BMI body mass index, FGR fetal growth restriction. ***p<0.001, ****p<0.0001; a FGR male versus AGA male; b FGR female versus AGA female.
